# Gender Differences for the Relative Age Effect on Physical Skills and Emotional Intelligence in Child Volleyball and Soccer Athletes

**DOI:** 10.3390/jfmk9040244

**Published:** 2024-11-20

**Authors:** Juan D. Ávila-Martínez, Michael A. Castro-Malaver, Boryi A. Becerra-Patiño, Juliana Varón-Murcia, Stefania Cárdenas-Contreras, José Pino-Ortega

**Affiliations:** 1 Sports and Physical Activity Sciences, Faculty of Physical Education, National Pedagogical University, Bogotá 111166, Colombia; jdavilam@upn.edu.co (J.D.Á.-M.); macastrom@upn.edu.co (M.A.C.-M.); 2 Faculty of Physical Education, National Pedagogical University, Bogotá 111166, Colombia; jjvaronm@upn.edu.co (J.V.-M.); scardenasc@upn.edu.co (S.C.-C.); 3 Management and Pedagogy of Physical Activity and Sport (GPAFD), Faculty of Physical Education, National Pedagogical University, Bogotá 111166, Colombia; 4 Faculty of Sports Sciences, University of Murcia, 30720 Santiago de la Ribera, Spain; josepinoortega@um.es

**Keywords:** evaluation, youth volleyball, youth soccer, athlete development, age grouping, skills, relative age effect

## Abstract

**Background/Objectives**. The goal of this study was to assess the relative age effect and its relationship with the development of physical abilities and emotional intelligence in Colombian child athletes according to gender and the sport practiced. **Methods**. A cross-sectional correlational design was used. A total of 135 Colombian soccer and volleyball athletes, 62 boys and 73 girls, voluntarily participated with an average age of 13.25 ± 0.59 years. Physical skills such as SJ, CMJ, CMJA, 5, 10, and 15 m speed, CODS agility, and emotional intelligence were evaluated. **Results**. Statistically significant differences were found in the mood variables between Q1 and Q4 (*p* = 0.047, ηp^2^ = 0.08) and Q2 and Q4 (*p* = 0.035, ηp^2^ = 0.08). These differences are also present in the interpersonal variable between Q1 and Q4 (*p* = 0.003, ηp^2^ = 0.12), Q2 and Q4 (*p* = 0.009, ηp^2^ = 0.12), and, finally, in the total emotional quotient between Q1 and Q4 (*p* = 0.013, ηp^2^ = 0.13), Q2 and Q3 (*p* = 0.024, ηp^2^ = 0.13), and Q2 and Q4 (*p* = 0.005, ηp^2^ = 0.13). **Conclusions**. Based on the findings of this research, it can be concluded that the relative age effect appears to be sensitive to CODS agility, 15 m speed, and SJ power variables concerning gender and sport. Jump height for CMJ concerning gender and CMJA jump concerning sport were considered. Emotional intelligence appears to be a variable sensitive to the relative age effect.

## 1. Introduction

Over the past 20 years, changes in school, social, and performance education have led to significant growth in sports, transitioning from recreational to high-performance sports [1]. Various factors, including the athletes’ financial situation, the age at which they start their sports journey, and competition or playing experience, are considered in the study of this phenomenon [2]. The age at which individuals start sports or intensive training influences talent selection and detection [1]. Athletes’ selection becomes problematic when deciding which ones participate in national representation programs [3]. Although some processes have been developed under the relative age effect (RAE) approach and others from biological maturation, it is necessary to recognize that there is no “gold standard” to help determine the processes of sports talent as they depend on interindividual characteristics, contextual factors, sport-specific characteristics, and long-term preparation processes according to Sweeney et al. [4]. This is reflected in the findings of the study conducted by Bezuglov et al. [5] when they determined that, although the evaluated footballers were more mature than late players (*p* < 0.001), in the end, there were no significant differences between them for variables related to strength, speed, or specific sports skills. Likewise, in another study with volleyball players, it was determined that there were no differences in physiological markers according to the RAE [6]. However, recent research has defined that age and maturation are indicators to consider in youth soccer and are influenced by gender and athletes’ competitive levels [7]. On the other hand, some studies have defined that the RAE generates bias in talent selection due to an inequality of opportunities in initial categories [8]. According to [9], the long-term goals of sports programs are to promote talents toward a specialty, developing crucial factors such as physical, technical, tactical, and psychological qualities connected with genetic variables and maturation development (biological age).

Therefore, an athlete born in the early months of the year can compete against one born later, leading to identified variability in biological age, i.e., differences in maturation among individuals of the same cohort [10]. Other studies determined a selection bias in RAE and maturation in football, suggesting that both processes should be analyzed independently [11,12]. Thus, chronological, biological, and cognitive age constitute RAE. However, based on biological and chronological age, three classifications can be distinguished: (i) children with early or premature maturation, developing before the present age; (ii) the development of individuals according to their age, with the peak acceleration occurring in boys at fourteen years and in girls at twelve; and (iii) late developments concerning the subject’s chronological age, as their biological age may be up to a year less than their chronological age [13].

Brown [14] emphasized that individuals in the late maturation stage are characterized by low stature but with a normal growth speed. These differences are crucial in studying the RAE. It can be evaluated by specific quartiles: 1 January to 31 March, 1 April to 30 June, 1 July to 30 September, and 1 October to 31 December [15,16]. In the same category, a chronological difference of 11 months could exist, generating differences in the physical and anthropometric conditions of individuals. RAE has studied playing positions in women’s soccer [17] as well as the influence of competitive-level and playing positions in young players [18]. Similarly, studies have focused on identifying and developing sports talents [19], the study of levels of sports experience in young soccer players [20], and the relationship between relative age, maturation, anthropometry, and physical fitness in young soccer players [21].

The systematic review by Sarmento et al. [19] concludes that studies focused on evaluating psychological and environmental factors that may affect talent development in soccer are needed. On the other hand, RAE has been studied for its effect on anthropometric and physiological characteristics in young volleyball players [22], vertical jump performance, and its effect on playing position and competitive levels [23], and the differences in position concerning physical and physiological characteristics [24]. Thus, soccer and volleyball are team cooperation–opposition sports with certain similarities in their game actions (jumps, direction changes, accelerations, decelerations, etc.), where multiple physical [25] and emotional [26] capabilities are manifested. The most relevant physical capacities in soccer and volleyball are speed, strength, and agility [27]. However, the emotional manifestation of athletes is crucial for improving the initiation and adherence to sports practice [28]. Emotions are a relevant indicator of sports performance [29], as they relate to perceptual, cognitive, motivational, and motor expression processes. This demonstrates the importance of research to further understand the existing differences among players about the RAE based on psychological variables, such as emotional intelligence (EI).

It has been studied that feelings and decisions could regulate the manifestation of sports performance [30], and these are closely related to moods and emotions [31]. Along these same lines, there is a meta-analytic study that determined the majority of profile mood scales (POMS) are predictors of performance in a wide variety of sports that are based on athletic performance [32]. These findings reaffirm the importance of the mood variable in sports and its link with the evaluation of EI. In this regard, the influence of EI on highly competitive sports has been studied [33] and its relationship with age and subjective well-being [34]. Ultimately, the cognitive aspect of an athlete is essential for their development; however, to date, no studies have addressed EI with RAE, as research has focused on comparing the physical, anthropometric, technical, and tactical characteristics of athletes without considering the athlete’s emotional aspect. A recent study on the analysis of RAE in sports determined that there is a greater scientific production of studies that study men (74.26%), while the production of male–female studies (20.46%) and women is low (7.01%) [35]. Another study addressed gender differences in preferences for team sports in adolescents, highlighting that the sports with the greatest preference are soccer and volleyball [36], which highlights the importance of making comparisons between sports based on the concept of gender. To our knowledge, no previous study evaluated the RAE on physical abilities and EI in young soccer and volleyball athletes in relation to gender. The present study aims to address this research gap. For this reason, the objective of the present study was to assess the RAE and its relationship with the development of physical abilities and EI in Colombian child athletes according to gender and the sport practiced.

## 2. Materials and Methods

### 2.1. Design

A cross-sectional correlational study design was used. Under the described experimental design and taking a type I error of 5% and a nominal power of 80%, the sample size was determined by means of the partial Eta2 statistic, taking as a reference the detection of small differences between quartiles, controlling for sport and gender in a significant way (Eta2 < 0.1). Below is the detail of the sample size for different values of Eta2. According to the given conditions, at least a sample size of 103 participants was required for the study; in the end, 135 participants were taken.

The research was woven through a series of organized, systematic, secure, and reliable processes, ensuring the integrity of all participants. Various criteria [37], characteristics, and protocols were considered for selection, evaluation, and data collection. Each athlete provided assent, and after the objectives, the scope and procedures of the study were thoroughly explained, and parents gave informed consent for voluntary participation. Each procedure followed principles outlined in the Declaration of Helsinki [38], and it adhered to resolution 8430 of the Ministry of Health of Colombia [39], declaring the study as low risk according to Colombian regulations based on non-invasive procedures. The study received approval from the National Pedagogical University’s ethics committee (340ETIC-2024).

### 2.2. Sample

The evaluated sample consisted of 135 Colombian athletes, including 62 boys and 73 girls, with an average age of 13.25 ± 0.59 years, a body mass of 51.10 ± 8.96 kg, and a height of 160.94 ± 8.57 cm. They were assessed for female soccer (*n*: 33), male soccer (*n*: 33), female volleyball (*n*: 40), and male volleyball (*n*: 29). Inclusion criteria were as follows: (i) absence of any injury, pathology, or surgery preventing participation; (ii) assent and informed consent signed by both the athlete and parents; (iii) age within the range of 12 to 13 years; (iv) completion of all tests; (v) engagement in volleyball and soccer; and (vi) a minimum of two years of experience in the corresponding sport, especially for familiarization and specificity of the actions performed such as jumps, CODS agility, and speed. Table 1 shows the basic characteristics of the sample evaluated.

### 2.3. Instruments

For weight determination, an Omron scale (Kyoto, Japan) with an accuracy of 0.1 kg was employed. The selected method for strength evaluation through the Squat Jump (SJ), Counter Movement Jump (CMJ), and Countermovement Jump with Arm Swing (CMJA) jumps was the mobile application My Jump 2, recording the video by camera, demonstrating a high intraclass correlation coefficient (0.997, *p* < 0.001), Bland–Altman bias = 1.1 ± 0.5 cm, *p* < 0.001, and validity for height r = 0.995, *p* < 0.001 [40,41]. CODS agility was assessed through the iPhone application COD-Timer, showing a total time measurement (r = 0.964; 95% confidence interval (CI) = 0.95–1.00; standard error of the estimate = 0.03 s; *p* < 0.001) [42]. To measure running mechanics, the iPhone application Runmatic was employed, showing a correlation coefficient (r = 0.94–0.99, *p* < 0.001) [43]. For EI evaluation, The Bar-On EQ-i: YV was used, specifically the 60-item version, considering inconsistency indices in each response [44]. The test demonstrated reliability through Cronbach’s alpha coefficient for the total scale of 0.76, and reliability indices for each factor ranged from 0.63 to 0.80 [45].

### 2.4. Procedure

Various protocols were used for the evaluation of physical capacities and EI. The evaluation was divided into four moments: (i) female volleyball; (ii) male volleyball; (iii) male soccer; and (iv) female soccer.

Each moment also spanned four days for the assessment of all variables, each separated by 48 h after the previous training session or competition match. On the first day, basic anthropometric measurements, including body mass and height, were taken. For the evaluation of physical capacities, the My Jump Lab application recorded the necessary information based on leg length (cm), trochanter length to the ground (cm), and squat length with a 90° angle. The second day involved the assessment of SJ, CMJ, and CMJA jumps [46]. The third day included speed tests at 5, 10, and 15 m, along with CODS agility. Finally, on the fourth day, an EI evaluation was conducted [44]. Each physical evaluation was performed by four researchers with a minimum of three months of experience using the My Jump Lab mobile application. In the application of The Bar-On Emotional Quotient Inventory: Youth Version (Bar-On EQ-i: YV), each of the four researchers was present to address any arising concerns. All data were collected under standard environmental conditions regarding time (4:00 to 6:00 pm) and temperature (between 15° and 22°) from August to October 2023. Each athlete was summoned 15 min before the start of training to undergo an 8 min warm-up protocol, which was standardized to include joint movements and lateral and frontal displacements. The facilities used were the soccer and volleyball fields regularly utilized by clubs for their training.

### 2.5. Statistical Analysis

Data analysis was conducted by study groups, initially involving a descriptive analysis divided into univariate, bivariate, and multivariate analyses. For quantitative variables, the central tendency was determined by the mean, dispersion by the standard deviation, and position measures by analyzing extremes and quartiles. In bivariate descriptive analysis, relationships between sets of variables were sought. It was necessary to classify variables into qualitative and quantitative, where qualitative variables included the year, quartile, sport, and gender, and a nested variable named the year quartile. In this sense, bivariate descriptive analysis was conducted in three sections.

For two qualitative variables, contingency tables were used. In the case of two quantitative variables, Pearson’s correlation was employed, and for one qualitative and one quantitative variable, univariate analysis was replicated, discriminating by category. For inferential analysis, considering the structure of groups in the variables, multivariate analysis of variance was utilized [47], using Pillai’s trace statistic [48], with prior suitability analysis (univariate and multivariate outlier analysis, univariate and multivariate normality, multicollinearity, and covariance homogeneity). In the case of finding a significant RAE effect, post hoc tests were applied to identify variables and age quartiles with a significant effect. Estimates of effect size for the main effects were calculated using partial eta squared (ηp^2^), interpreted as follows: 0.01—small; 0.06—medium; ≥0.14—large [49]. Finally, the analyses were executed using software version 4.1.0.

## 3. Results

The following Table 2, Table 3, Table 4 and Table 5 show the description of the variables from the average and standard deviation in relation to the evaluated variables of strength, speed, CODS agility, and EI, as well as for gender, sport, quartile, and year of birth.

### 3.1. Significant Differences for the CODS Agility Profile According to RAE

For the CODS agility variable, large significant differences were found for the right COD deficit between Q1 and Q4 (*p* = 0.009, ηp^2^ = 0.16), Q2 and Q3 (*p* = 0.049, ηp^2^ = 0.16), and Q2 and Q4 (*p* = 0.000, ηp^2^ = 0.16). Similarly, for the right time variable, significant differences were found in Q1 and Q4 (*p* = 0.009, ηp^2^ = 0.18), Q2 and Q3 (*p* = 0.018, ηp^2^ = 0.18), and Q2 and Q4 (*p* = 0.000, ηp^2^ = 0.18), and all these differences had a large effect size. On the contrary, in the right average speed variable, the effect size was medium; nevertheless, significant evidence was found when comparing Q1 and Q4 (*p* = 0.006, ηp^2^ = 0.12) and Q2 and Q4 (*p* = 0.000, ηp^2^ = 0.12).

Statistically significant differences were evident in the left COD left deficit variable in Q1 and Q4 (*p* = 0.026, ηp^2^ = 0.13), Q2 and Q3 (*p* = 0.044, ηp^2^ = 0.13), and Q2 and Q4 (*p* = 0.003, ηp^2^ = 0.13); this variable had a medium effect size. For the left time variable, the same significant differences were found as in the left COD deficit variable, except for the Q1 and Q4 comparison (*p* = 0.027, ηp^2^ = 0.14). Finally, for left average speed, large significant differences were found in Q1 and Q4 (*p* = 0.022, ηp^2^ = 0.13), Q2 and Q3 (*p* = 0.034, ηp^2^ = 0.13), and Q2 and Q4 (*p* = 0.002, ηp^2^ = 0.13) (Table 6).

When comparing the right COD deficit and right time with the sport, there were significant differences in volleyball players, with both variables showing differences between Q2 and Q4 (*p* = 0.022), all with a large effect size. Large significant differences were found in certain quartiles when comparing CODS agility variables with the female gender, such as right COD deficit in Q2 and Q4 (*p* = 0.006, ηp^2^ = 0.29), left COD deficit in Q2 and Q4 (*p* = 0.031, ηp^2^ = 0.29), right time in Q2 and Q3 (*p* = 0.044, ηp^2^ = 0.29), as well as right time between Q2 and Q4 (*p* = 0.006, ηp^2^ = 0.29) and left time in Q2 and Q4 (*p* = 0.03, ηp^2^ = 0.29). Finally, in the right average speed variable, differences were found between Q2 and Q4 (*p* = 0.005, ηp^2^ = 0.20), and in the left average speed variable, differences were between Q2 and Q4 (*p* = 0.03, ηp^2^= 0.30).

### 3.2. Significant Differences in the EI Profile According to the RAE

For EI, significant differences were observed in the mood variable between Q1 and Q4 (*p* = 0.047, ηp^2^ = 0.08) and Q2 and Q4 (*p* = 0.035, ηp^2^ = 0.08). Likewise, there were large significant differences in the interpersonal variable between Q1 and Q4 (*p* = 0.003, ηp^2^ = 0.12) and Q2 and Q4 (*p* = 0.009, ηp^2^ = 0.12). Finally, in the total emotional quotient, large significant differences were observed between Q1 and Q4 (*p* = 0.013, ηp^2^ = 0.13), Q2 and Q3 (*p* = 0.024, ηp^2^ = 0.13), and Q2 and Q4 (*p* = 0.005, ηp^2^ = 0.13). For the positive impression variable, significant differences were found between Q2 and Q3 (*p* = 0.024, ηp^2^ = 0.06), but with a small effect size (Table 7).

In the mood variable, large significant differences in the EI profile for soccer were found between Q1 and Q4 (*p* = 0.004, ηp^2^ = 0.05), Q2 and Q4 (*p* = 0.003, ηp^2^ = 0.05), and Q3 and Q4 (*p* = 0.006, ηp^2^ = 0.05). The interpersonal variable showed that large significant differences in the EI profile about soccer were found in Q1 and Q4 (*p* = 0.004, ηp^2^ = 0.01) and Q2 and Q4 (*p* = 0.014, ηp^2^ = 0.01). Additionally, there were large significant differences in the EI profile for soccer that were found in the interpersonal variable in Q1 and Q3 (*p* = 0.001) and Q2 and Q3 (*p* = 0.033). Regarding the total emotional quotient, there was a medium effect size with large significant differences in Q1 and Q4 (*p* = 0.012, ηp^2^ = 0.13) and Q2 and Q4 (*p* = 0.037, ηp^2^ = 0.13) (Table 8).

Continuing with the EI profile for the sport of volleyball, there were large significant differences in the interpersonal variable between Q2 and Q3 (*p* = 0.035, ηp^2^ = 0.01) and Q2 and Q4 (*p* = 0.019, ηp^2^ = 0.01). In the total emotional quotient variable, there were significant differences between Q2 and Q3 (*p* = 0.037). When comparing the EI profile by gender, significant differences for females were evident in the mood variable between Q1 and Q4 (*p* = 0.047) and the interpersonal variable in Q1 and Q4 (*p* = 0.018). Regarding the total emotional quotient, there were significant differences in Q1 and Q4 (*p* = 0.038, ηp^2^ = 0.21) and Q2 and Q4 (*p* = 0.015, ηp^2^ = 0.21) along with a large effect size. There were significant differences when comparing men with the adaptability variable (*p* = 0.049, ηp^2^ = 0.11), with a medium effect size in this comparison.

### 3.3. Significant Differences for the SJ Profile

There were significant differences between sports for the profile exhibited by SJ jump variables and controlling for age quartiles (*p* = 0.029). For the power variable, Q2 in volleyball achieved higher results than Q3 in soccer, which had the lowest result for this variable. There were large significant differences between genders for the SJ jump profile by quartiles (*p* = 0.000), where male athletes showed greater strength in the jump than female athletes, especially in Q4, where males had higher results.

### 3.4. Significant Differences for the CMJ Profile

**Figure 1:**
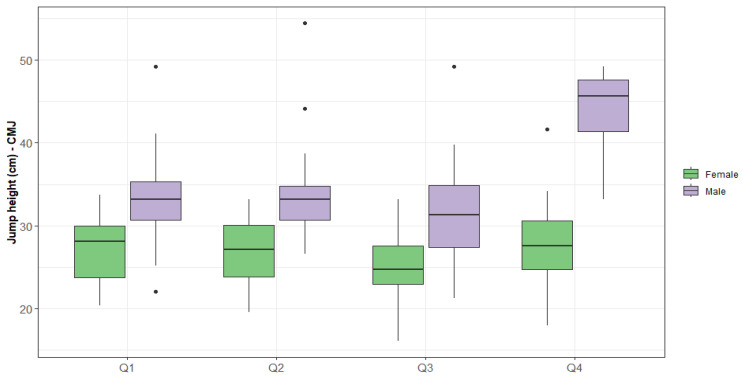
CMJ jump height by quartile and gender.

Within the CMJ jump profile, there were relevant differences between genders for the profile exhibited by CMJ jump variables, controlling for age quartiles (*p* = 0.000). Considering the analysis, both the female and male gender in Q4 had the highest results for the jump height variable, while Q3 had the lowest values for this variable (Figure 1).

### 3.5. Significant Differences for the CMJA Profile

**Figure 2:**
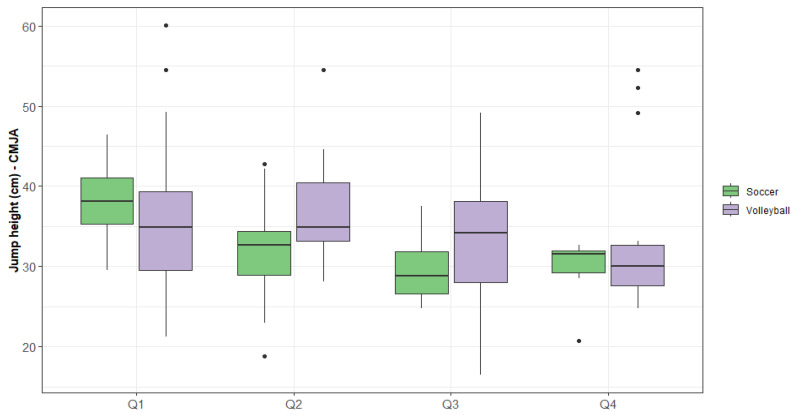
CMJA jump height by quartile and sport.

For the CMJA profile controlled by quartiles, large significant differences between sports were evident (*p* = 0.023). In the jump height variable, Q2 volleyball players obtained higher results than Q4 soccer players. Contrary to this, Q1 in soccer achieved superior results to Q4 in volleyball. Regarding gender, large significant differences were found (*p* = 0.000), as male athletes in Q1 had a higher jump height than female athletes in Q4 (Figure 2).

### 3.6. Significant Differences in Speed Profile

There were large significant differences between sports in speed variables when controlling for quartiles (*p* = 0.000). In the 15 m speed variable, athletes from all quartiles in soccer had shorter times compared to volleyball athletes. Specifically, in the first quartile (Q1) of soccer, the time was notably shorter than in the fourth quartile (Q4) of volleyball.

## 4. Discussion

The purpose of this study was to assess the RAE and its relationship with the development of physical capacities and EI in team sports (soccer and volleyball) and gender in Colombian athletes born in 2009 and 2010. Based on the findings of this research, it can be concluded that the RAE appears to be sensitive to CODS agility, 15 m speed, and SJ power variables concerning gender and sport. The jump height for CMJ concerning gender and the CMJA jump concerning sport were also considered. When comparing these CODS agility variables by gender, females exhibited the most significant differences in the right COD deficit for Q2 and Q4, the right time in Q2 and Q4, and the right average speed variable between Q2 and Q4. Likewise, EI appears to be a variable sensitive to the relative age effect.

The impact of relative age has been studied from a broad perspective, emphasizing its significance, with limited exploration regarding its connection to EI. This study serves as a precedent for understanding this phenomenon from physical and emotional perspectives, delving into how this effect manifests in these capacities.

Following Smith et al. [50], the RAE is observed between the 12 and 15 years of age range in female sports. This aligns with our findings, indicating the presence of this effect in both female and male team sports, which is consistent with other studies [51,52]. Similarities were found when comparing genders, with males achieving better results due to significant differences. This phenomenon was present for CODS agility variables in females (*p* < 0.006).

CODS agility showed significant differences within the same year, revealing that athletes born in the first two quartiles performed better than those in the last quartiles. Similar results were found by [53], who conducted a 30 m agility test. Moreover, [54] found significant differences between genders for speed (20 m) and CODS agility, corroborating the findings of this study. In line with the study by Ek et al. [55], the comparison between sports for the countermovement jump did not show significant differences between quartiles of relative age, expressing *p* < 0.12. However, contrary to this, the current study determined differences between sports for countermovement jumps in SJ and CMJA regarding power and jump height. The study by Sandercock et al. [56], which found better results for athletes born in November in cardiorespiratory fitness, strength, and power, did not align with the current study’s results. In this study, soccer players born between January and March (Q1) achieved superior results compared to Q4 in volleyball. When considering gender, significant differences (*p* = 0.000) were found, with male athletes in Q1 achieving greater jump heights than female athletes in Q4.

According to Yoshimura et al. [57], athletes who practiced volleyball had better results for the SJ jump profile when compared to other sports. These results did not correspond to those of the present study, where it was shown that for the profile exhibited by the SJ jumping variables, athletes who practiced soccer had better results compared to those who played volleyball (*p* = 0.029). 

Warneke et al. [58] found significant differences between ages (10 and 14 years) for speed measured at 10 m and 20 m, stating that older participants achieved better results. However, the current research revealed no significant differences in speed measured at 5, 10, and 15 m when comparing birth years (2009 and 2010). Still, there were differences in speed variables concerning quartiles (*p* = 0.000). For the 15 m speed variable, athletes in all quartiles of soccer exhibited shorter times compared to those in volleyball. Significant differences between quartiles of relative age were found in the EI profile, with *p* < 0.001, indicating that athletes in Q1 and Q2 achieved better results in the total emotional quotient (eta squared 0.13). This contradicted [59], who found that relative age was an inconsistent predictor of perceptual–cognitive variable performance.

The study conducted by Soylu et al. [60] concluded that EI levels vary depending on age and positioning on the field, thus determining that psychological and physiological responses are equally necessary for football’s specific performance. A similar case to the results of the present study was identified, where differences in physical capacities were found in relation to SJ, CMJ, CMJA, CODS agility, and especially with EI variables related to interpersonal characteristics, intrapersonal characteristics, positive impression, total emotional quotient, and mood.

The variable related to EI in female soccer players allowed the specific character of the athlete concerning the specific demands of the sport to be determined, concluding that EI has a significant impact on human adaptability and the effectiveness of psychosocial functioning, so its study allows development paths for athletes to be identified [61]. With this, and following the findings of the present study, all evaluated EI variables turned out to be significant in response to the RAE. With this consideration, the results obtained offer the opportunity to propose specific actions that favor long-term sports development based on physical and psychological variables.

A study developed to understand EI and the practice of physical sports activity determined that children who practiced organized sports showed better skills in intrapersonal, interpersonal, adaptability, mood states, and EI. Similarly, studies on gender and age found that girls have higher levels of EI in the interpersonal dimension and mood states, while boys have higher levels of interpersonal intelligence and stress management [62]. In that sense, the results of the present study, by recognizing physical abilities and EI but in response to the RAE, found differences in mood states between Q1 and Q4, the interpersonal dimension between Q1 and Q4, and the emotional quotient for Q1–Q4 and Q2–Q4. Meanwhile, in the case of men, there were differences in adaptability. This corroborates the importance of recognizing psychological variables such as EI for evaluating athletes’ sports performance based on gender, sport, and year of birth.

Maghsoudipour et al. [63] used the Bar-On EI test on 20-year-old track and field athletes, concluding that EI, especially interpersonal skills, empathy, and independence, influence athlete performance. Similar results were found in the current study, where EI was proven to be sensitive to the RAE. On the contrary, [64] found no significant correlation between EI and sports performance (*p* > 0.05). In contrast, the current study determined that EI and physical abilities are sensitive to the RAE in soccer and volleyball players. There are differences in some EI components (empathy, interpersonal relationships, and independence) that were found to correlate with reaction time, similar to the current study’s findings, where significant differences were found for EI variables (mood, positive impression, interpersonal, intrapersonal, and total emotional quotient) along with CODS agility test times for both the left and right sides.

Additionally, this study identified, through the BarOn test, that sports practice is influenced by stress management and intrapersonal emotions. This indicates that athletes with higher scores in these categories could influence their results and achievements. This aligns with the current study’s results, demonstrating that better stress control and expression with others fall within the first and second quartiles of both branches, even when represented in sports. These findings are consistent with the results reported by Kopp et al. [33], who emphasized the use of EI in studies for performance comparisons between sports and performance levels, often based on birth years or nested quartiles, as observed in the current study concerning gender.

The consideration of EI can help establish the implementation of sports development programs since it is a determining variable to achieve results in sports performance [65]. This consideration is corroborated by the findings of the present study when considering that EI is a variable sensitive to RAE in young soccer and volleyball players.

### Limitations and Future Directions

The main limitations are related to the number of Q4 athletes born in 2010 for both sports and genders. Based on the results of this study, some recommendations were identified for future lines of research. Firstly, the influence of EI on sports performance in various sports continues to be a challenge for the different areas that contribute to the long-term sports process, especially sports psychology. On the other hand, it is suggested that future research can incorporate the study of EI together with other variables associated with sports performance, such as physical and technical–tactical capacities.

It is suggested that studies continue evaluating and developing scientific production comparing physical and, especially, emotional capacities in a larger population and different sports. Given the limited investigations related to EI and considering the findings of this research, statistically significant differences were evidenced in EI concerning the RAE. Finally, it is necessary to note that these findings can help enhance the understanding of other variables influencing sports performance in relation to EI. Longitudinal studies with the variables investigated in this research and the integration of technical–tactical variables are suggested to determine the changes athletes experience in their sports performance.

## 5. Conclusions

The EI seems to be a variable sensitive to the RAE. Significant differences were found for nested quartiles, gender, and sport in interpersonal variables for Q1 and Q4, Q2 and Q4, the total emotional quotient between Q2 and Q4, mood in Q1 and Q4, Q2 and Q4, and positive impression between Q2 and Q3.

In conclusion, this study focused on considering physical (SJ, CMJ, CMJA, CODS agility, and speed) and psychological (EI) athletes according to gender variables to understand the effect of RAE on soccer and volleyball players, demonstrating that mainly emotional variables are more sensitive to this effect for the evaluated athletes.

## Figures and Tables

**Table 1:** Bivariate characterization of height, body mass, gender, sport, and quartile.

Variable		Height		Body Mass	
Gender		Female	Male	Female	Male
Sport	Volleyball	158.09 ± 5.70	169.63 ± 8.57	51.86 ± 9.45	56.88 ± 9.95
	Soccer	156.53 ± 6.69	161.18 ± 7.78	48.36 ± 6.91	47.83 ± 6.58
Year		2009			
Quartile	Q1	157.91 ± 8.52	170.77 ± 9.50	50.11 ± 5.80	57.10 ± 9.55
	Q2	157.25 ± 5.23	163.35 ± 9.29	52.95 ± 5.05	48.41 ± 6.94
	Q3	158.73 ± 4.71	164.91 ± 8.03	58.08 ± 12.3	52.34 ± 7.58
	Q4	157.21 ± 3.40	176.22 ± 4.81	49.08 ± 5.40	57.68 ± 4.48
Year		2010			
Quartile	Q1	155.75 ± 7.77	166.56 ± 7.39	47.72 ± 5.56	53.19 ± 9.45
	Q2	158.11 ± 5.66	164.36 ± 6.79	45.29 ± 3.19	53.52 ± 11.9
	Q3	156.23 ± 7.88	156.23 ± 8.61	53.75 ± 13.5	45.57 ± 8.43

**Table 2:** Bivariate characterization of SJ, CMJ, and CMJA in response to gender, the sport practiced and quartile of athletes.

Variable		JH SJ		JH CMJ		JH CMJA	
Gender		Female	Men	Female	Men	Female	Men
Sport	Voleyball	24.98 ± 4.1	25.66 ± 4.1	25.66 ± 4.1	37.19 ± 8.0	29.33 ± 4.6	43.15 ± 7.8
	Soccer	25.75 ± 4.7	27.91 ± 4.9	27.91 ± 4.9	31.39 ± 4.3	29.85 ± 5.3	35.40 ± 4.9
Year	2009						
Q	Q1	26.12 ± 2.5	36.65 ± 6.1	27.02 ± 3.4	37.72 ± 6.1	30.57 ± 4.0	43.70 ± 7.8
	Q2	26.72 ± 4.4	30.71 ± 5.6	27.20 ± 4.4	33.28 ± 4.5	29.47 ± 4.6	37.55 ± 7.4
	Q3	22.88 ± 5.8	34.78 ± 5.5	25.39 ± 5.4	36.89 ± 6.0	29.18 ± 6.2	41.64 ± 4.2
	Q4	26.03 ± 2.8	39.10 ± 5.9	27.27 ± 6.0	43.40 ± 7.1	29.50 ± 3.1	46.89 ± 10.1
Year	2010						
Q	Q1	25.91 ± 4.6	29.65 ± 2.1	27.09 ± 4.6	32.13 ± 6.3	33.01 ± 7.5	38.79 ± 6.0
	Q2	24.15 ± 4.1	32.28 ± 7.1	26.34 ± 4.1	34.78 ± 7.5	29.64 ± 4.3	38.80 ± 6.5
	Q3	23.78 ± 4.6	25.82 ± 3.9	24.88 ± 4.1	27.56 ± 3.8	27.41 ± 4.3	31.60 ± 4.0
	Q4	27.78 ± 6.3	/	29.7	/	27.70 ± 4.0	/

JH SJ: Jump height squat jump; JH CMJ: jump height countermovement jump; JH CMJA: jump height countermovement jump with arms; and Q: quartile.

**Table 3:** Bivariate characterization of speed.

Var		0–5 m		5–10 m		10–15 m		Total 15 m	
Gender		Female	Men	Female	Men	Female	Men	Female	Men
S	V	1.70 ± 0.2	1.51 ± 0.3	0.96 ± 0.0	0.82 ± 0.0	0.89 ± 0.1	0.79 ± 0.0	3.55 ± 0.3	3.11 ± 0.4
	So	1.51 ± 0.2	1.37 ± 0.2	0.86 ± 0.0	0.82 ± 0.0	0.79 ± 0.0	0.76 ± 0.0	3.18 ± 0.2	2.95 ± 0.3
Year		2009							
Q	Q1	1.69 ± 0.3	1.45 ± 0.2	0.91 ± 0.0	0.81 ± 0.0	0.93 ± 0.0	0.73 ± 0.0	3.53 ± 0.4	2.99 ± 0.3
	Q2	1.63 ± 0.2	1.39 ± 0.2	0.89 ± 0.0	0.80 ± 0.0	0.81 ± 0.0	0.76 ± 0.0	3.35 ± 0.2	2.92 ± 0.2
	Q3	1.59 ± 0.4	1.39 ± 0.3	0.89 ± 0.1	0.76 ± 0.0	0.80 ± 0.0	0.78 ± 0.0	3.28 ± 0.4	2.95 ± 0.4
	Q4	1.67 ± 0.2	1.51 ± 0.4	0.97 ± 0.0	0.81 ± 0.1	0.84 ± 0.1	0.77 ± 0.0	3.49 ± 0.4	3.09 ± 0.5
Year		2010							
Q	Q1	1.65 ± 0.2	1.52 ± 0.3	0.92 ± 0.1	0.85 ± 0.0	0.86 ± 0.1	0.78 ± 0.0	3.43 ± 0.3	3.16 ± 0.3
	Q2	1.42 ± 0.2	1.41 ± 0.2	0.87 ± 0.0	0.82 ± 0.0	0.80 ± 0.0	0.75 ± 0.0	3.10 ± 0.3	2.96 ± 0.3
	Q3	1.63 ± 0.3	1.39 ± 0.2	0.94 ± 0.0	0.86 ± 0.0	0.87 ± 0.1	0.83 ± 0.0	3.45 ± 0.4	3.08 ± 0.3
	Q4	1.64 ± 0.3	/	0.93 ± 0.0	/	0.90 ± 0.1	/	3.47 ± 0.4	/

Var: variable; S: sport; V: volleyball; So; soccer; and Q: quartile.

**Table 4:** Bivariate characterization of CODS agility.

Var			CODS Agility Right		CODS Agility Left	
Gender		Female	Female	Men	Female	Men
S	V	0.96 ± 0.0	20.7 ± 1.82	18.1 ± 1.21	20.9 ± 1.70	18.2 ± 1.29
	So	0.86 ± 0.0	18.4 ± 1.01	16.8 ± 1.12	18.4 ± 1.12	16.9 ± 1.05
Year		2009				
Q	Q1	0.91 ± 0.0	19.8 ± 2.11	17.0 ± 1.21	20.1 ± 2.26	16.8 ± 1.11
	Q2	0.89 ± 0.0	18.5 ± 0.77	17.0 ± 0.97	18.4 ± 0.97	17.0 ± 0.87
	Q3	0.89 ± 0.1	19.7 ± 1.65	18.0 ± 1.69	19.4 ± 1.75	18.0 ± 1.26
	Q4	0.97 ± 0.0	20.7 ± 2.43	17.7 ± 0.52	20.6 ± 2.21	17.6 ± 0.53
Year		2010				
Q	Q1	0.92 ± 0.1	19.1 ± 1.49	17.6 ± 1.15	19.4 ± 1.87	17.9 ± 1.21
	Q2	0.87 ± 0.0	18.8 ± 1.28	16.9 ± 1.40	19.5 ± 1.90	17.1 ± 1.65
	Q3	0.94 ± 0.0	20.1 ± 2.02	17.9 ± 1.71	20.2 ± 1.78	18.2 ± 1.59
	Q4	0.93 ± 0.0	20.9 ± 1.75	/	21.2 ± 2.03	/

Var: variable; S: sport; V: volleyball; So; soccer; and Q: quartile.

**Table 5:** Characterization of EI variables.

Var		Total Emotional Quotient		Intrapersonal		Interpersonal		Adaptability		Stress Management		Mood	
Gen		Female	Men	Female	Men	Female	Men	Female	Men	Female	Men	Female	Men
S	V	176 ± 21.0	196 ± 21.3	93 ± 15.6	96 ± 11.5	102 ± 9.2	105 ± 12.5	102 ± 20.1	118 ± 16.9	89 ± 15.79	92 ± 15.03	99 ± 18.4	100 ± 16.9
	So	181 ± 17.6	202 ± 18.3	92 ± 13.0	100 ± 11.2	100 ± 13.4	105 ± 13.9	103 ± 17.2	114 ± 22.7	92 ± 13.17	91 ± 12.42	104 ± 14.8	111 ± 9.9
Year		2009											
Q	Q1	189 ± 26.6	215 ± 16.2	100 ± 22.5	105 ± 8.8	105 ± 6.5	107 ± 10.2	110 ± 25.0	120 ± 12.8	94 ± 7.63	99 ± 13.99	111 ± 32.6	118 ± 7.5
	Q2	184 ± 10.8	209 ± 19.3	93 ± 10.5	1007.5	102 ± 11.2	106 ± 10.3	105 ± 19.8	124 ± 23.6	94 ± 14.73	95 ± 17.50	107 ± 12.8	111 ± 12.3
	Q3	174 ± 20.4	192 ± 23.3	82 ± 12.8	98 ± 11.1	106 ± 14.8	99 ± 15.8	103 ± 18.5	108 ± 20.5	87 ± 17.30	94 ± 18.28	101 ± 14.9	105 ± 14.5
	Q4	169 ± 15.7	206 ± 27.5	89 ± 12.8	99 ± 12.5	97 ± 10.4	97 ± 17.4	97 ± 16.7	135 ± 11.8	87 ± 13.45	94 ± 10.27	95 ± 14.68	105 ± 19.8
Year		2010											
Q	Q1	182 ± 14.9	191 ± 13.7	96 ± 13.1	95 ± 15.1	106 ± 9.5	107 ± 12.5	97 ± 19.2	104 ± 22.2	95 ± 14.58	89 ± 11.13	105 ± 11.4	100 ± 15.3
	Q2	187 ± 22.3	201 ± 18.2	100 ± 12.0	99 ± 14.0	104 ± 13.9	111 ± 11.6	113 ± 18.2	122 ± 15.6	90 ± 11.56	87 ± 11.32	103 ± 15.2	105 ± 13.3
	Q3	173 ± 24.2	189 ± 17.1	89 ± 16.3	97 ± 7.79	100 ± 7.8	99 ± 12.0	100 ± 16.8	112 ± 17.4	86 ± 20.07	89 ± 11.80	100 ± 16.2	100 ± 16.2
	Q4	163 ± 5.1	/	95 ± 11.9	/	89 ± 11.9	/	102 ± 18.3	/	86 ± 11.62	/	86 ± 16.51	/

Var: variable; Gen: gender; S: sport; V: volleyball; So; soccer; Q: quartile; and EI: emotional intelligence.

**Table 6:** Results of the CODS agility profile according to the RAE by quartile.

**Variable**	**Group 1**	**Group 2**	***p*. Adj**	**Effect Size**	**CI (95%)**
COD right deficit	Q1	Q4	0.009 **	0.16	[0.07, 1.00]
COD right deficit	Q2	Q3	0.049 *	0.16	[0.07, 1.00]
COD right deficit	Q2	Q4	0.000 ***	0.16	[0.07, 1.00]
Right time	Q1	Q4	0.009 **	0.18	[0.08, 1.00]
Right time	Q2	Q3	0.018 *	0.18	[0.08, 1.00]
Right time	Q2	Q4	0.000 ***	0.18	[0.08, 1.00]
Right average speed	Q1	Q4	0.006 **	0.12	[0.03, 1.00]
Right average speed	Q2	Q4	0.000 ***	0.12	[0.03, 1.00]
**Variable**	**Group 1**	**Group 2**	***p*. Adj**	**Effect Size**	**CI (95%)**
COD left deficit	Q1	Q4	0.026 *	0.13	[0.04, 1.00]
COD left deficit	Q2	Q3	0.044 *	0.13	[0.04, 1.00]
COD left deficit	Q2	Q4	0.003 **	0.13	[0.04, 1.00]
Left time	Q1	Q4	0.027 *	0.14	[0.05, 1.00]
Left time	Q2	Q3	0.044 *	0.14	[0.05, 1.00]
Left time	Q2	Q4	0.003 **	0.14	[0.05, 1.00]
Left average speed	Q1	Q4	0.022 *	0.13	[0.04, 1.00]
Left average speed	Q2	Q3	0.034 *	0.13	[0.04, 1.00]
Left average speed	Q2	Q4	0.002 **	0.13	[0.04, 1.00]

*, significant difference at *p* < 0.05, **, significant difference at *p* < 0.01, and ***, significant difference at *p* < 0.001.

**Table 7:** Results of EI profile according to RAE.

Variable	Group 1	Group 2	*p*. Adj	Effect Size	CI (95%)
Mood	Q1	Q4	0.047 *	0.08	[0.01, 1.00]
Mood	Q2	Q4	0.035 *	0.08	[0.01, 1.00]
Positive impression	Q2	Q3	0.024 *	0.06	[0.00, 1.00]
Interpersonal	Q1	Q4	0.003 **	0.12	[0.04, 1.00]
Interpersonal	Q2	Q4	0.009 **	0.12	[0.04, 1.00]
Total emotional quotient	Q1	Q4	0.013 *	0.13	[0.04, 1.00]
Total emotional quotient	Q2	Q3	0.024 *	0.13	[0.04, 1.00]
Total emotional quotient	Q2	Q4	0.005 **	0.13	[0.04, 1.00]

*, significant difference at *p* < 0.05, **, significant difference at *p* < 0.01.

**Table 8:** EI profile results according to RAE by sport (soccer).

Variable	Group 1	Group 2	*p*. Adj	Effect Size	CI (95%)
Mood	Q1	Q4	0.004 **	0.05	[0.01, 1.00]
Mood	Q2	Q4	0.003 **	0.05	[0.01, 1.00]
Mood	Q3	Q4	0.006 **	0.05	[0.01, 1.00]
Interpersonal	Q1	Q4	0.004 **	0.01	[0.00, 1.00]
Interpersonal	Q2	Q4	0.014 *	0.01	[0.00, 1.00]
Intrapersonal	Q1	Q3	0.001 ***	-	[0.00, 1.00]
Intrapersonal	Q2	Q3	0.033 *	-	[0.00, 1.00]
Total emotional quotient	Q1	Q4	0.012 *	0.13	[0.04, 1.00]
Total emotional quotient	Q2	Q4	0.037 *	0.13	[0.04, 1.00]

*, significant difference at *p* < 0.05, **, significant difference at *p* < 0.01, and ***, significant difference at *p* < 0.001.

## Data Availability

The raw data supporting the conclusions of this article will be made available by the authors without undue reservation.
